# Epidemiology and biological plausibility in assessing causality

**DOI:** 10.1097/EE9.0000000000000177

**Published:** 2021-11-11

**Authors:** David A. Savitz

**Affiliations:** Department of Epidemiology, Brown University School of Public Health, Providence, Rhode Island.

## Introduction

One of the well-accepted principles of epidemiology is the need to draw upon ancillary evidence from biological research in the selection of topics to pursue, design of studies, and especially, the interpretation of the results. In environmental epidemiology, understanding the biological pathways by which the exposure of concern may affect health often has a great value in framing research questions and guiding the studies that are done but calls for deeper reflection of how that ancillary biological information should (and should not) be used.

Biological evidence of potential health harm may motivate epidemiologic studies and help to guide exposure assessment to maximize the likelihood of identifying an etiologic relationship if one is present. Decisions regarding exposure aggregation (lumping or splitting), duration of exposure, the timing of exposure in relation to disease occurrence, exploration of dose-response patterns (thresholds and ceilings), and other chemical and physical features of exposure to be examined in epidemiologic studies benefit from drawing on knowledge from pertinent biological research. Similarly, the choice of specific disease entities should be informed by knowledge of biological mechanisms in the analogous decisions regarding grouping, timing of onset, distinctive features of the disease (e.g., subsets of cancer with a shared etiology), and clinical manifestations. Markers of susceptibility that could result in effect-modification may be gleaned from biological research as well as candidate confounders. The product of this knowledge drawn from work done in other fields, if considered in advance, is an enhanced ability to design and conduct epidemiologic studies in which the measure of association is most likely to identify any causal effects that are present, that is, more valid studies. If positive or negative associations are found, they would be seen as concordant with expectations based on biology and if null associations are found, this would provide meaningful evidence that the plausible etiologic relationship is not likely to be present. But the dividing line between using biological evidence to optimize the design of studies and the use of biological evidence to render a verdict on the *validity* of the study calls for a closer look.

The consideration of biological plausibility in the interpretation of the study results, as advocated by Sir Austin Bradford Hill^1^ raises some concerns when considering why measured associations may or may not reflect a causal effect. To the extent that the epidemiologic research is informed by sound biological insights, we will benefit from having focused on the most pertinent exposure and disease measures, minimizing exposure and disease misclassification, and isolating the most highly susceptible subgroups. Once we have gleaned all that we can from ancillary biological research on the topic, however, the epidemiologic study must stand on its merits in order to approximate the causal effect of interest, driven solely by freedom from biases. As elucidated below, the presence (or absence) of biological information does not independently influence the validity of the epidemiologic study that was conducted, which is determined solely by the usual methodological considerations. There is no validity bonus at the end for the epidemiologic studies because other lines of evidence point in the same direction nor are validity points deducted because of the lack of such support.

If valuable information from biological research is not taken into account in the epidemiologic study due to lack of knowledge at the time the study was undertaken or because the biological research came later, validity will suffer. In that sense, the biological evidence does help to inform us as to whether misclassification is likely to have been present, for example, or whether underlying effect modification has not been taken into account. But this reconciliation of what is known from the varying lines of evidence can only take place when the research from multiple disciplines is compiled and then integrated for a global assessment of causality. With the comprehensive body of relevant research, we may find converging evidence from the varying lines of evidence supporting causality or that the epidemiologic research provides a flawed assessment of the etiologic process once evidence from other disciplines is taken into account. The aggregation of knowledge from the multiple contributing disciplines is needed for a fully informed assessment of causality, rather than looking to epidemiology alone or epidemiology interpreted through biology.

## Implications of alternative perspectives on the biological context for epidemiology

Epidemiology alone is generally not the sole basis for causal inference and subsequent policy decisions, nor should it be. Such evaluations call for a comprehensive assessment of all relevant evidence, including epidemiology, biological research, and other relevant lines of investigation. The system used by the International Agency for Research on Cancer makes that strategy quite explicit,^2^ with a clear role for toxicologic and mechanistic evidence as contributors to causal assessment. Note that the mechanistic evidence is drawn upon not *through its effect on interpreting the epidemiologic evidence* but *as an independent contributor* to the ultimate assignment that integrates all sources of relevant evidence. The need for rigorous evaluation of epidemiologic methods remains the basis for assessing epidemiology’s contribution to that assignment. The key distinction is as indicated in the diagram below:

**Figure:**
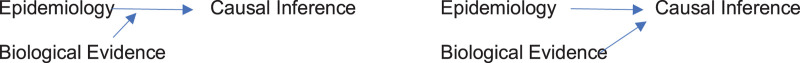


The diagram on the left suggests that the value of the epidemiology to causal inference is modified by ancillary evidence whereas the diagram on the right indicates each is independently contributing to causal inference. While it may seem like a minor semantic distinction whether the biological evidence is brought to bear as a means of *interpreting the epidemiologic evidence* or as an *independent contributor to causal inference*, there are a number of potentially important consequences of that choice. It is worth noting that Professor Hill’s original article did not provide considerations in the *interpretation of epidemiologic evidence* (as they have come to be used) but rather for making an overall judgment regarding *whether environmental exposures cause disease*.^1^ Reasons for favoring the use of the biological evidence directly rather than as an adjunct to the interpretation of epidemiology are noted below.

## Validity of epidemiologic research is solely a function of study methods

The goal of etiologic epidemiology research is to generate measures of association that approximate the causal effect of interest. The extent to which these deviate from one another is solely a function of the susceptibility of the study to biases, that is, confounding, measurement error, selection bias, or random error. Logically, a spurious positive result is not in error because it does not make biological sense but because some bias is operating to indicate an association when no underlying causal effect is in fact present. Likewise, an erroneous null finding in an epidemiologic study must be attributed to methodologic limitations if a causal effect is in fact present. As noted in a commentary on the relationship between the GRADE approach and Bradford Hill criteria, biological plausibility does not contribute to the assessment of the validity of the research.^3^ While ancillary evidence from other disciplines may help to inform how effective the epidemiologic study was in addressing the most important or relevant potential effects, the study’s results can only be judged based on whether or not the resulting measure of association does or does not reflect the causal impact of the exposure and disease that were addressed, that is, its internal validity.

Some years ago, epidemiologic studies suggested a possible effect of electromagnetic fields from power lines on the risk of cancer.^4–6^ A frequent criticism of biophysicists and engineers was that the epidemiologic findings must be wrong because they run counter to well-established findings from these other disciplines, but this is logically impossible. If the epidemiologic studies identified spurious positive associations when no causal effect was, in fact, present, then the problem had to lie within the epidemiologic studies, for example, selection bias or measurement error. The source of error cannot be attributed to contradictory evidence from other lines of research.

## Epidemiologists are generally incapable of accurately interpreting biological evidence

While epidemiologists may be conversant with the biological research on the etiologic relationship that they are studying, without deep subject matter knowledge it is very difficult to fully appreciate the validity and relevance of research from these other disciplines. The methods and implications of various types of biological research are no less subtle than their counterparts in epidemiologic studies, subject to critical evaluation by experts in those fields. Just as is the case for epidemiology, experimental studies in the laboratory have varying degrees of both internal validity and applicability to the study of humans in the real world. This goes beyond the familiar observation that exposure levels or modes of administration differ, and includes nuances of species differences in metabolism, laboratory artifacts, and validity of outcome measurement.

A common challenge is to distinguish between biological responses indicative of normal variation or adaptation to environmental stressors versus indicators of disease processes, which can be quite subtle. Just as those lacking expertise in epidemiology often interpret our work simplistically and fail to appreciate the nuances of methods and implications for validity, the same problems are inevitable when epidemiologists attempt to interpret research in biologically complex disciplines in which they have minimal training and experience. In collective assessments of evidence such as those that are routinely undertaken by the International Agency for Research on Cancer or the US National Academy of Sciences, Engineering, and Medicine, the full range of needed disciplinary experts is engaged and the informed evaluation of each relevant line of evidence is essential to integrate them.

## Rigorous and pertinent biological research contributes independent of epidemiology

There is an implicit downgrading of biological evidence when it is used only to assist in the interpretation of epidemiologic evidence. Toxicology can and often does contribute significantly to judgments of causality and regulatory decisions to protect human health. Increasingly, as understanding of biological interactions between environmental exposures and humans expands, mechanistic studies have come to be recognized as important contributors to an overall assessment of human health risks.^2^ The “bottom line” judgment of causality is based on the entire array of relevant research in proportion to their informativeness in both absolute terms and relative to one another. Since epidemiologic studies address the relevant species (humans) in the environments of interest (the real world), there is a temptation to treat those results as providing the ultimate answers regarding human health, but the power of various forms of biological research is pertinent and has complementary methodologic strengths and weaknesses relative to epidemiology. Ultimately, inferences regarding causality and subsequent policy decisions require a level of generalization and abstraction that goes beyond any particular study or line of research.

## Concluding comments

The considerations offered by Sir Austin Bradford Hill over 50 years ago provide a diverse menu of ideas that were offered to judge environmental causes of disease but contribute to the interpretation of epidemiologic evidence in several ways.^1^ Some are pertinent to assessing the potential for bias as the basis for observed associations rather than a causal effect—magnitude of effect, dose-response, and experimentation concern potential confounding and specificity indirectly addresses measurement error (e.g., response bias). But the consideration of biological plausibility, unquestionably relevant to causal inference, is external to epidemiologic studies. To treat biological plausibility as a tool to aid in the interpretation of epidemiology overstates its value in assessing the validity of epidemiologic evidence and understates its real value in assessing causality.

## Conflicts of interest statement

The author declares that he has no conflicts of interest with regard to the content of this repor
